# *Euonymus alatus*-Derived Extracellular Vesicles Show Immunomodulatory Effects in an Inflammatory Thyroid Follicular Cell Model: Insights from Multi-Omics Analysis

**DOI:** 10.3390/biomedicines14102201

**Published:** 2026-09-29

**Authors:** Yang Li, Yanqiong He, Yi Guo, Chuan Hua, Xin-He Zuo, Man Tian, Yong Zhao

**Affiliations:** 1 Thyroid Center, Hubei Provincial Hospital of Traditional Chinese Medicine, Wuhan 430061, China; liyang@hbhtcm.hbucm.edu.cn (Y.L.); heyanqiong@hbhtcm.com (Y.H.);; 2 Affiliated Hospital of Hubei University of Chinese Medicine, Hubei Provincial Hospital of Traditional Chinese Medicine, Wuhan 430061, China; 3 Department of Nuclear Medicine, Hubei Provincial Hospital of Traditional Chinese Medicine, Wuhan 430061, China; 4 Hubei Shi Zhen Laboratory, Wuhan 430061, China

**Keywords:** *Euonymus alatus*, extracellular vesicles, autoimmune thyroiditis, Nthy-ori-3-1 cells, proteomics, RNA-seq

## Abstract

**Objectives**: To extract and identify extracellular vesicles (EVs) derived from the traditional Chinese herb *Euonymus alatus* (EA), characterize their molecular profiles using multi-omics analysis, and assess their regulatory effects and potentially associated signaling pathways in an interferon-γ (IFN-γ)-stimulated inflammatory thyroid follicular cell model mimicking key inflammatory features of autoimmune thyroiditis (AIT). **Methods**: Differential centrifugation combined with density gradient centrifugation was used to purify EA-derived EVs, and the isolates were characterized using a BCA protein assay, transmission electron microscopy (TEM), nanoparticle tracking analysis (NTA), and Western blotting (WB). Molecular constituents were examined via label-free proteomics and RNA-seq. Meanwhile, Nthy-ori-3-1 cells were exposed to IFN-γ to establish an inflammatory thyroid follicular cell model; these activated cells were subsequently treated with the purified EVs (EV group). The supernatants were collected and the levels of interleukin-1β (IL-1β) and interleukin-18 (IL-18) were determined by ELISA. Comparative transcriptomic and proteomic profiling was employed to identify differentially expressed molecules and related signaling pathways between the model and the EV group. **Results**: EA-derived EVs were successfully isolated and showed typical EV characteristics. Proteomics identified 3607 proteins enriched in metabolic, immune, and antioxidant processes, while RNA-seq generated 72.16 million clean reads and revealed genes enriched in metabolic and immune pathways. In the IFN-γ-stimulated Nthy-ori-3-1 cells, ELISA results showed that EV treatment significantly reduced the secretion of IL-1β and IL-18 compared to the model group. EV intervention resulted in 138 differential proteins (42 upregulated, 96 downregulated) and 262 differential genes (81 upregulated, 181 downregulated). GO analysis revealed significant enrichment in terms related to inflammatory response, immune response, and regulation of oxidative stress. KEGG pathway analysis demonstrated enrichment in the IL-17, NOD-like receptor, and PPAR signaling pathways, as well as ferroptosis, and Wnt pathway (unique for upregulated genes). **Conclusions**: EA-derived EVs are rich in molecules that regulate metabolic pathways and immune responses. They may ameliorate inflammation in this inflammatory thyroid follicular cell model, with multi-omics analyses revealing correlative enrichment of multiple signaling pathways. These findings provide preliminary experimental evidence and novel candidate targets for AIT treatment.

## 1. Introduction

Autoimmune thyroiditis (AIT), predominantly Hashimoto’s thyroiditis, represents the commonest autoimmune disorder affecting the thyroid gland, serologically defined by circulating thyroid autoantibodies [1]. The pathogenesis of AIT is driven by lymphocytic infiltration, ongoing inflammation, tissue fibrosis, and damage to thyroid cells [2], which are promoted by pro-inflammatory mediators, notably interferon-γ (IFN-γ) and tumor necrosis factor-α (TNF-α) [3,4]. These destructive events eventually lead to primary hypothyroidism [5]. Beyond thyroid dysfunction, AIT imposes a substantial multisystem problem: it significantly heightens the risks of adverse obstetric outcomes (e.g., spontaneous abortion and preterm delivery) [6], and may affect neurocognitive development in offspring [7]. Moreover, the autoimmune inflammatory state has been linked to neuropsychiatric manifestations [8], cardiovascular disorders [9], and comorbid autoimmune conditions such as vitiligo [10], autoimmune gastritis [11], and rheumatoid arthritis [12]. Given these far-reaching clinical implications, there is a compelling need to advance research on AIT prevention and treatment. Currently, therapeutic options for AIT remain largely symptomatic. Hormone replacement with levothyroxine is prescribed only after overt hypothyroidism develops, yet this regimen does not counteract the underlying autoimmune inflammation [5]. In addition, improper dosing may raise the risk of cardiac arrhythmias and osteoporosis [1]. Other options, including glucocorticoids, selenium, or vitamin D supplementation, have failed to demonstrate consistent efficacy or satisfactory safety records [13,14]. These limitations highlight the need to discover novel, mechanism-based therapeutic agents for AIT.

According to the Shennong Ben Cao Jing (Shennong’s Classic of Materia Medica), *Euonymus alatus* (EA) has long been used to alleviate inflammatory disorders [15,16], promote blood circulation, dissipate stasis, resolve masses, and reduce edema [17]. Modern phytochemical investigations have identified flavonoids, terpenoids, and other bioactive constituents in EA, which exhibit anti-inflammatory, antioxidant, anti-fibrotic, and immunomodulatory activities [18,19]. Based on these pharmacological properties of EA and the well-established Nthy-ori-3-1 cell model of thyroid epithelial inflammatory injury, we sought to investigate the therapeutic potential of EA in AIT.

Plant-derived extracellular vesicles (EVs), nanovesicles carrying abundant proteins and nucleic acids, are increasingly recognized as key regulators of immune responses [20,21]. In recent years, plant-derived EVs have attracted considerable interest as a frontier in the modernization of traditional Chinese medicine [22,23]. Their effect on the autoimmune process is gradually being substantiated; for example, EVs from Petasites japonicus were reported to upregulate surface molecules (CD80, CD86, MHC-I, and MHC-II) on dendritic cells and to promote Th1-skewing cytokine secretion [24]. These properties prompted us to hypothesize that EA-derived EVs might function as bioactive nanocarriers and exert therapeutic actions in AIT.

Given the limitations of current therapies for AIT and the promising potential of plant-derived EVs, the present study was designed with four objectives: (1) to isolate and characterize EA-derived EVs; (2) to analyze their protein and RNA contents through label-free proteomics and RNA-seq; (3) to evaluate their regulatory effects on IFN-γ-stimulated Nthy-ori-3-1 cells (an inflammatory thyroid follicular cell model mimicking key inflammatory features of AIT); and (4) to identify potentially associated signaling pathways linked to their anti-inflammatory and immunoregulatory effects via integrated multi-omics profiling.

## 2. Materials and Methods

### 2.1. Experimental Materials

Samples: Fresh winged twigs of *Euonymus alatus* (EA) (collected in Bijie, Guizhou Province, China; botanically authenticated by Hubei Provincial Hospital of Traditional Chinese Medicine).

Cell line: Nthy-ori-3-1 (human thyroid follicular epithelial), Procell, CL-0817, Wuhan, China.

Reagents: Interferon-γ (IFN-γ, PeproTech, 300-02, Cranbury, NJ, USA; specific activity ≥ 2 × 10^7^ IU/mg). Sucrose (Solarbio, S8271, Beijing, China); BCA Protein Assay Kit (Beyotime, P0009, Shanghai, China); CCK-8 assay Kit (Yisheng Biotechnology, 40203ES60, Shanghai, china); CD9 polyclonal antibody (Proteintech, 20597-1-AP, Wuhan Sanying, Wuhan, China); CD81 polyclonal antibody (Proteintech, 27855-1-AP, Wuhan Sanying, China); IL-1β ELISA Kit (Elabscience, E-HSEL-H0001, Wuhan, China); IL-18 ELISA Kit (Elabscience, CQH007, Wuhan, China); Polystyrene microspheres (Thermo Fisher, 3100A, Fremont, CA, USA); RPMI-1640 medium (Gibco, 11875085, Grand Island, NY, USA); fetal bovine serum (HyClone, Cytiva, SH30088, Logan, UT, USA); and TRIzol reagent (Invitrogen, 15596018, Carlsbad, CA, USA).

Equipment: Multimode microplate reader (PerkinElmer, VICTOR Nivo, Waltham, MA, USA); ultracentrifuge (Beckman, Optima L-100XP, Brea, CA, USA); transmission electron microscope (TEM, JEOL, JEM1400, Tokyo, Japan); nanoparticle tracking analyzer (NTA, Particle Metrix, Zetaview-PMX120-Z, Inning am Ammersee, Germany); nucleic acid electrophoresis apparatus (Junyi-Dongfang, JY300C, Beijing, China); gel imaging system (Junyi-Dongfang, JY04S-3C, Beijing, China); Orbitrap Astral mass spectrometer (Thermo Fisher (Bremen) GmbH, Bremen, Germany); and Illumina sequencing platform(Illumina, Inc., San Diego, CA, USA).

### 2.2. Extraction and Identification of EA-Derived Extracellular Vesicles (EVs)

Extraction: Fresh winged shoots of EA served as the raw material for EV isolation. After grinding in PBS, the homogenate was centrifuged by differential steps at 4 °C (1000× *g* for 10 min, followed by 3000× *g* for 20 min and 10,000× *g* for 30 min). The resulting supernatant was then ultracentrifuged at 100,000× *g* for 1 h, and the pellet obtained was resuspended in PBS. After 15–60% sucrose density gradient centrifugation (150,000× *g* for 2 h), the middle layer (30–45%) was collected, diluted with an equal volume of PBS, and centrifuged at 150,000× *g* for 1 h. The pellet was resuspended in PBS to generate a purified EV suspension [25].

Identification: Protein concentration was assessed using a standard curve constructed with BSA, and the optical density (OD) was measured at 562 nm. The sample was dropped onto a copper grid, negatively stained with 3% phosphotungstic acid, and observed under a TEM. The sample was diluted with PBS for NTA of particle-size distribution and concentration; the instrument was pre-calibrated with 100 nm polystyrene beads. Exosomal markers CD9 and CD81 were detected by WB (primary antibody dilution ratio: 1:1000; secondary antibody dilution ratio: 1:5000). Rat-serum-derived extracellular vesicles were included as a positive control for WB detection.

### 2.3. Multi-Omics Analysis of EA-Derived EVs

EV multi-omics analyses were performed on one biological preparation of EA-derived EVs for exploratory descriptive cargo characterization.

Proteomics: EV proteins were processed for digestion: lysed, reduced and alkylated (TCEP/CAA), digested overnight with trypsin (1:50 enzyme/substrate ratio, 37 °C), and desalted. LC-MS/MS analysis was then carried out on an Orbitrap Astral instrument (DIA mode). DIA-NN (v1.8) was used for protein identification and quantification. All identified proteins were annotated against the Gene Ontology (GO) and Kyoto Encyclopedia of Genes and Genomes (KEGG) databases using species-specific annotation resources, followed by enrichment analysis [26].

RNA-seq: Total EV RNA was isolated using TRIzol reagent and assessed for integrity. mRNA was enriched with Oligo(dT) beads for cDNA library construction and sequenced on an Illumina platform. Raw reads were quality-checked by FastQC (v0.11.5) and processed with fastp (v0.23.2). Clean reads were mapped to the EA reference genome with Hisat2 (v2.2.1); uniquely mapped reads were retained and PCR duplicates were removed prior to transcript quantification using featureCounts (v2.0.1) [27]. All detected EV transcripts were annotated against GO and KEGG databases using species-specific annotation resources, followed by enrichment analysis.

### 2.4. Cell Experiments and Intervention of Inflammatory Model

Cell culture and grouping: Nthy-ori-3-1 cells were maintained in RPMI-1640 supplemented with 10% fetal bovine serum at 37 °C with 5% CO_2_. An *in vitro* inflammatory cell model mimicking AIT was established by stimulation with IFN-γ, in the concentration of 500 IU/mL, based on the previous studies [28,29,30]. To screen for the optimal EA-derived EV concentration that exhibited minimal cytotoxicity while conferring protection against IFN-γ-induced injury, a CCK-8 assay was performed. Nthy-ori-3-1 cells were exposed to either IFN-γ alone or IFN-γ combined with increasing doses (10, 50, or 100 μg/mL) of EA-derived EVs for 24, 36, or 48 h. Based on the CCK-8 data, a concentration of 50 μg/mL was chosen for the following experiments. The experiment comprised four groups, as detailed below:

▪ Control: Nthy-ori-3-1 cells were cultured under normal conditions for 36 h.
▪ Model (IFN-γ): Cells were continuously stimulated with 500 IU/mL IFN-γ for 36 h.
▪ Intervention (IFN-γ + EA-EVs): Cells were continuously stimulated with 500 IU/mL IFN-γ for 36 h, with 50 μg/mL EA-derived EVs co-administered during the final 12 h.
▪ EV control (EA-EVs): Cells were maintained under normal culture conditions for 24 h, followed by treatment with 50 μg/mL EA-derived EVs for an additional 12 h (total 36 h).

The anti-inflammatory potential of EA-derived EVs was evaluated by quantifying IL-1β and IL-18 in culture supernatants via ELISA kits. All measurements were carried out in duplicate.

Multi-omics analysis for cellular samples: Cellular proteomic and transcriptomic analyses were performed on the Model and Intervention groups, each with *n* = 3 independent biological replicates. Cell lysates were subjected to protein extraction, reduction-alkylation, trypsin digestion and desalting, followed by LC-MS/MS analysis on an Orbitrap Astral mass spectrometer in DIA mode. Proteins were identified and quantified with DIA-NN (v1.8) against the UniProt human reference protein database; after normalization, differentially abundant proteins were screened. Total RNA was isolated using TRIzol reagent for constructing mRNA-enriched Illumina cDNA libraries. Transcripts were mapped to the *Homo sapiens* hg38_gencode_v40 reference genome using Hisat2 (v2.2.1), and quantified with featureCounts (v2.0.1); differentially expressed transcripts were identified using DESeq2 (v1.34.0). Differential proteins and transcripts were annotated against GO and KEGG databases using species-specific annotation resources, followed by enrichment analysis.

### 2.5. Statistical and Enrichment Analyses

GraphPad Prism 9.0 was used for statistical analysis. CCK-8 and ELISA data were normally distributed, and quantitative data were expressed as mean ± SD. CCK-8 data were analyzed by two-way ANOVA with treatment and time as factors, followed by Tukey’s multiple comparison test; ELISA data were analyzed by one-way ANOVA followed by Tukey’s post hoc test. Statistical significance was set at *p* < 0.05.

GO and KEGG enrichment analyses were performed using clusterProfiler (v4.2.2) with Fisher’s exact test, and the Benjamini–Hochberg method was applied for multiple-testing correction; pathways with FDR-adjusted *p*-value < 0.05 were considered significantly enriched. For omics profiling of EA-derived EVs, enrichment was conducted on all proteins/transcripts detected in the one biological preparation against species-specific annotated gene sets, and the results were interpreted descriptively. For cellular omics, differentially expressed features were defined as |FC| ≥ 2 and FDR-adjusted *p*-value < 0.05; enrichment was conducted on differentially expressed transcripts and differentially abundant proteins (*n* = 3 independent biological replicates) against the corresponding full human gene/protein background. The top-20 enriched GO and KEGG terms were visualized based on adjusted *p*-values and gene/protein-count percentages [31].

## 3. Results

### 3.1. Extraction and Identification of EA-Derived EVs

#### 3.1.1. Protein Concentration

BCA assay showed that the protein concentration of EA-derived EVs was 0.548 mg/mL (average OD562 value: 0.568), which met the requirements of subsequent experiments (Figure 1a).

#### 3.1.2. Morphology and Particle Size

TEM imaging showed that the obtained particles were predominantly cup-shaped or hemispherical, measuring 30–150 nm in diameter (Figure 1b), which matched the well-established EV morphology. No obvious impurity contamination was observed, indicating that EVs with intact morphology were successfully isolated and obtained.

NTA further revealed that the particles had a mean diameter of 151.9 nm, the main peak was at 121.7 nm, and the particle concentration was 6.1 × 10^10^ particles/mL (Figure 1c). These results further confirmed that the extracted vesicles met the core characteristics of EVs in terms of particle size and concentration, with high purity.

#### 3.1.3. Marker Detection

Western blotting (WB) results showed that specific bands of the classic animal exosomal markers CD9 (theoretical molecular weight: approximately 25 kDa) and CD81 (theoretical molecular weight: approximately 23 kDa) were not detected in EA-derived EV samples (Figure 1d(i)). In contrast, CD9/CD81 bands were successfully detected in the positive control (rat serum exosomes, Figure 1d(ii)). This result suggested that the markers of plant-derived EVs may have species specificity and are different from those of animal exosomes.

### 3.2. Multi-Omics Analysis of EA-Derived EVs

#### 3.2.1. Proteomic Analysis

The length distribution of identified peptides peaked at 9–11 amino acids (Figure 2a), consistent with the expected pattern of tryptic digestion. Most of the detected proteins were characterized by 1–3 peptides per protein (Figure 2b), a typical profile for high-confidence proteomic datasets. Overall, 16,699 unique peptides corresponding to 3607 proteins were successfully identified (Figure 2c), confirming the high quality of the data for subsequent analyses.

GO enrichment analysis demonstrated that, apart from being localized to photosynthesis-related organelles (chloroplasts and thylakoids), the proteome of EA-derived EVs was predominantly associated with categories related to small molecule metabolism, organic acid metabolism, and carboxylic acid metabolism (Figure 3a). For the biological process (BP) category, the terms with the highest number of associated genes included reproduction, negative/positive regulation of biological process and immune process. In the molecular function (MF) category, the most highly represented terms were structural molecular activity, transporter activity, and antioxidant activity (Figure 3b).

KEGG enrichment analysis indicated that the proteome of EA-derived EVs was predominantly enriched in the ribosome pathway. The major enriched pathway types were metabolism-related pathways (e.g., glycolysis, oxidative phosphorylation, and carbon fixation), along with several signaling cascades, including the HIF-1 pathway (Figure 4a). In terms of functional classification, the proteins were primarily enriched in metabolic pathways and genetic information processing (Figure 4b).

#### 3.2.2. RNA-Seq Analysis

A total of 72,161,224 clean reads were obtained, with a clean read rate of 98.52% and a mapping rate of 81.02%. GO enrichment analysis revealed enrichment in cellular component (CC) terms such as ribosome- and thylakoid-related structures, as well as in MF terms including structural constituent of ribosome (Figure 5a). For the BP category, the terms with the largest number of associated genes included regulation of biological process, reproduction, and immune system process. The most highly represented MF terms were transcription regulator activity and transporter activity (Figure 5b).

The identified genes were found to be significantly enriched in KEGG pathways including ribosome and photosynthesis, dominated by metabolic pathways (e.g., carbon fixation and carbohydrate metabolism) and basic physiological pathways (e.g., ribosome biogenesis and photosynthesis) (Figure 6a). Functional classification analysis indicated primary enrichment in metabolism-related categories (including carbohydrate and energy metabolism) and genetic translation (Figure 6b).

### 3.3. Cell Experiments

#### 3.3.1. Viability of IFN-γ-Stimulated Nthy-Ori-3-1 Cells Following EA-Derived EV Treatments

A time-dependent decrease in cell viability was observed following IFN-γ stimulation, as compared with the untreated controls (Figure 7). This reduction was most pronounced at the 48-h time point, confirming the successful establishment of an inflammatory cell injury model. Notably, co-treatment with EVs markedly reversed the IFN-γ-induced decrease in cell viability. This protective effect was concentration-dependent, with the 50 μg/mL concentration demonstrating the most robust efficacy across all three time points (24, 36, and 48 h), restoring cell viability to levels comparable with or even slightly exceeding those of the control group. While the 10 μg/mL EV treatment partially improved cell viability, the effect was weaker than that of the 50 μg/mL dose. The highest concentration (100 μg/mL) also showed protection, but its efficacy was attenuated compared with the 50 μg/mL group, suggesting a potential plateau or subtle adverse effect at supraphysiological concentrations.

#### 3.3.2. EA-Derived EVs Inhibit IFN-γ-Induced Secretion of Pro-Inflammatory Cytokines in Nthy-Ori 3-1 Cells

A marked elevation in the secretion of both IL-1β and IL-18 was observed following IFN-γ stimulation, as compared with the control group (Figure 8), suggesting the onset of inflammatory activation.

Co-treatment with EVs (50 μg/mL) significantly lowered the IFN-γ-induced upregulation of IL-1β and IL-18. Meanwhile, treatment with EVs alone at the same concentration showed no significant effect on the basal cytokine levels compared with the control group, indicating that EVs themselves do not exert pro-inflammatory effects and that this inhibitory effect is specific to the IFN-γ-induced inflammatory context.

#### 3.3.3. Proteomic Analysis

A total of 138 differentially expressed proteins—42 showing increased expression and 96 showing decreased expression—were detected in the EV-treated group compared with the controls (Figure 9).

GO enrichment analysis indicated that these proteins were predominantly associated with regulation of inflammatory response, complement regulator activity, and tumor necrosis factor (TNF) receptor activity (Figure 10a), and the associated functional categories were largely clustered in regulation of molecular function (Figure 10b). When classified by expression direction, the downregulated proteins in AIT model cells after EV intervention were mainly linked to pathways governing inflammatory response, humoral immune response, proteolysis, protein processing, and response to external stimuli (Figure 10c). In contrast, the upregulated counterparts were enriched in processes involving smooth muscle cell proliferation, negative regulation of epidermis development, and negative regulation of oxidative stress-induced processes (Figure 10d).

KEGG pathway mapping of the differentially expressed proteins revealed significant enrichment in complement and coagulation cascades, and cytokine–cytokine receptor interaction (Figure 11a), with functional classifications concentrated in the immune system category (Figure 11b). The downregulated proteins were predominantly assigned to complement and coagulation cascades, the IL-17 signaling pathway, and ferroptosis (Figure 11c), while the upregulated proteins were mainly associated with the PI3K-Akt signaling pathway and apoptosis (Figure 11d).

#### 3.3.4. RNA-Seq Analysis

A total of 262 genes (81 upregulated and 181 downregulated) were identified in the EV-treated cells compared with the model group (Figure 12).

GO functional annotation of these genes showed enrichment in inflammatory response, immune response, and cellular response to tumor necrosis factor (Figure 13a), with functional classifications concentrated in immune system process and transporter activity (Figure 13b). Among the downregulated genes in EV-treated AIT model cells, enrichment was observed in inflammatory response, immune response, cytokine activity, cellular response to TNF/IL-1, type I interferon signaling pathway, and positive regulation of T cell migration (Figure 13c). Among the upregulated genes, enrichment was noted in cell differentiation, negative regulation of cell migration, and chemical synaptic transmission (Figure 13d).

KEGG enrichment mapping assigned the differentially expressed genes mainly to NOD-like receptor signaling, IL-17 signaling, and PPAR signaling (Figure 14a), with immune system, infectious diseases, and endocrine and metabolism as the predominant functional classes (Figure 14b). Among the downregulated genes in AIT model cells after EV intervention, enriched pathways included NOD-like receptor signaling, and IL-17 signaling (Figure 14c). Notably, among the upregulated genes, the Wnt signaling pathway showed the highest enrichment score (Figure 14d).

## 4. Discussion

This study investigated the biological properties of EVs derived from the traditional Chinese medicine EA and their interventional effects on an inflammatory thyroid follicular cell model mimicking AIT. By systematically characterizing their molecular composition and inferred functional features using multi-omics approaches, this work provides novel theoretical insights and a research basis for the modernization of traditional Chinese medicine-based therapy for AIT.

In this study, EVs derived from EA were isolated and comprehensively characterized, confirming their typical EV features. WB analysis did not detect the conventional mammalian exosomal markers CD9 or CD81, which is consistent with the species specificity of plant-derived exosomal markers and provides initial clues for future marker identification.

Multi-omics characterization of EA-derived EVs revealed their molecular features and functional orientation. Proteomic analysis revealed that the identified proteins were enriched in functional terms related to small molecule metabolism, immune system processes, and antioxidant activity, together with photosynthesis-associated organelles (chloroplasts and thylakoids). KEGG annotation of the same protein set assigned them predominantly to metabolism-related pathways (glycolysis and oxidative phosphorylation) and to signaling pathways such as HIF-1. In parallel, GO enrichment of the transcriptomic data linked the corresponding genes to metabolic routes, basic physiological processes, immune-related functions, and molecular terms covering transcription regulator and transporter activities. The proteins and RNAs enriched in EA-derived EVs are broadly consistent with the traditional efficacy and modern pharmacological studies of EA [16,18,19]. As natural carriers of bioactive molecules, EA-derived EVs are enriched in a large number of metabolism-regulating and immune-related proteins and RNAs, supporting their potential for “metabolic regulation-immune modulation” and multi-target regulatory effects in the AIT inflammatory microenvironment.

IFN-γ stimulation successfully induced an inflammatory phenotype. Compared with untreated controls, IFN-γ triggered a time-dependent decline in cell viability and markedly increased the secretion of IL-1β and IL-18, consistent with previous reports [29]. Multi-omics profiling of the cell model identified a set of differentially expressed molecules and associated signaling pathways between the intervention (IFN-γ + EA-EVs) and the model (IFN-γ) groups. At the proteomic level, GO enrichment revealed core functional categories encompassing regulation of inflammatory response, complement regulator activity, and TNF receptor activity. Among the downregulated proteins, enrichment was observed in pathways related to regulation of inflammatory response, humoral immune response, and proteolysis, whereas the upregulated proteins showed enrichment in smooth muscle cell proliferation and negative regulation of oxidative stress-induced processes. Of note, TNF receptor activity has been closely linked to HT pathogenesis, as its engagement promotes the secretion of pro-inflammatory cytokines (e.g., IFN-γ, IL-6) by infiltrating immune cells, aggravates follicular cell injury, and stimulates the production of thyroid autoantibodies [32,33]. KEGG analysis of the differentially expressed proteins revealed significant enrichment in complement and coagulation cascades, as well as cytokine–cytokine receptor interaction. Among these, downregulation of the complement and coagulation cascade has been associated with reduced immune complex-mediated tissue injury [34]. Additionally, the downregulation of the IL-17 signaling pathway—a pro-inflammatory axis highly expressed in AIT—may potentiate the anti-inflammatory effect of EA-derived EVs by interrupting a key step in the inflammatory cascade [35]. Consistently, ELISA quantification of culture supernatants showed that EV treatment significantly reduced IL-1β and IL-18 secretion in IFN-γ-stimulated Nthy-ori-3-1 cells compared to the model group, two well-recognized upstream inducers of IL-17 production and Th17 differentiation [36]. Moreover, suppression of the ferroptosis pathway may confer additional protective effects on thyroid follicular epithelial cells by limiting the accumulation of lipid peroxidation products [37]. These correlative omics findings indicate that EA-derived EVs may possess potential immunomodulatory and thyroprotective properties, suggestive of a multi-target regulatory profile. Notably, the hyperactivation of the PI3K/AKT axis [38] and apoptosis are both associated with the severity of AIT, and the observation that both are upregulated warrants further investigation in the context of EA-derived EV intervention.

Transcriptomic GO annotation showed that the differentially expressed genes were mainly enriched in inflammatory response, immune response, cytokine activity, and chemokine-mediated signaling. Specifically, the downregulated genes were involved in inflammatory and immune responses, chemokine activity, and type I interferon signaling, which may counteract IFN-γ-mediated inflammatory cascades. The upregulated genes were enriched in terms related to cell differentiation and negative regulation of cell migration, hinting that EA-derived EVs may help sustain the homeostasis of the local microenvironment of thyroid follicular cells. KEGG enrichment further linked these genes to NOD-like receptor, IL-17, PPAR, and Wnt signaling. The NOD-like receptor and IL-17 pathways [29,39], both central to autoimmune inflammation, were downregulated, which may reduce inflammatory factor release and immune cell activation. Among the upregulated pathways, the most pronounced enrichment was observed for the Wnt signaling pathway. Based on previous reports, Wnt signaling can facilitate Treg generation [40], restrain Th17 differentiation [41], and reduce IL-17-mediated inflammatory responses; it may also inhibit NF-κB activity [42] through the regulation of β-catenin nuclear transcription [43,44]. If these literature-described functions are operative within our experimental system, Wnt pathway activation may partially contribute to the integrated anti-inflammatory effects of EA-derived EVs.

Based on these enrichment observations, we tentatively propose a hypothetical regulatory framework. First, EA-derived EVs may suppress inflammatory initiation and amplification by attenuating IFN-γ and type I interferon signaling, as well as by downregulating the IL-17 and NOD-like receptor pathways, which could curtail cytokine release and immune cell recruitment and limit inflammatory infiltration. Second, they may facilitate the restoration of immune tolerance through Wnt-mediated Treg/Th17 rebalancing and suppression of humoral immune responses and TNF receptor activity, potentially reducing both cellular and humoral autoimmune attacks. Third, they appear to protect thyroid follicular cells, possibly by inhibiting ferroptosis. This putative integrated regulatory network aligns with the known traditional therapeutic properties of EA and suggests a multi-target regulatory profile of EA-derived EVs in AIT.

### Limitations

This study represents an initial attempt to identify metabolism-regulating and immune-related molecules in EA-derived EVs and to explore their possible effects on AIT. This study has several important limitations. First, no well-validated gold-standard markers for plant-derived EVs are currently available, so the biogenesis and sub-cellular origin of the isolated EA-derived EVs remain incompletely defined. Second, EV multi-omics analyses were performed on a single biological preparation; these descriptive enrichment results reflect only this batch of EV cargo and cannot be generalized. Third, functional assays were limited to an IFN-γ-stimulated Nthy-ori-3-1 cell model. This simplified in vitro system recapitulates thyroid epithelial inflammatory injury but lacks the immune-cell crosstalk characteristic of authentic AIT. Fourth, key pathways including IL-17, NOD-like receptor, Wnt and ferroptosis were inferred from correlative omics enrichment together with the published literature. Causal mechanistic conclusions cannot be established without targeted functional perturbation experiments. Future studies could address these issues by performing pathway-specific functional validation and further dissecting the molecular basis behind the observed pathway modulations.

## 5. Conclusions

In summary, EA-derived EVs were successfully isolated and characterized as typical extracellular vesicles, and multi-omics profiling revealed that they carry abundant metabolism-regulating and immune-related molecular cargo. In an IFN-γ-stimulated inflammatory thyroid follicular cell model mimicking AIT, EA-derived EV treatment reduced the secretion of the pro-inflammatory cytokines IL-1β and IL-18, and comparative multi-omics analyses identified differentially expressed proteins and transcripts enriched in several inflammation- and immunity-related pathways, including IL-17, NOD-like receptor, Wnt, and ferroptosis signaling. These pathway associations are correlative rather than causal, and functional perturbation experiments are required to verify the proposed pathway involvement. Overall, our findings provide preliminary experimental evidence and novel candidate targets for plant-derived EVs as a potential therapeutic strategy for AIT, which warrant further investigation in functional assays and in vivo models.

## Figures and Tables

**Figure 1:**
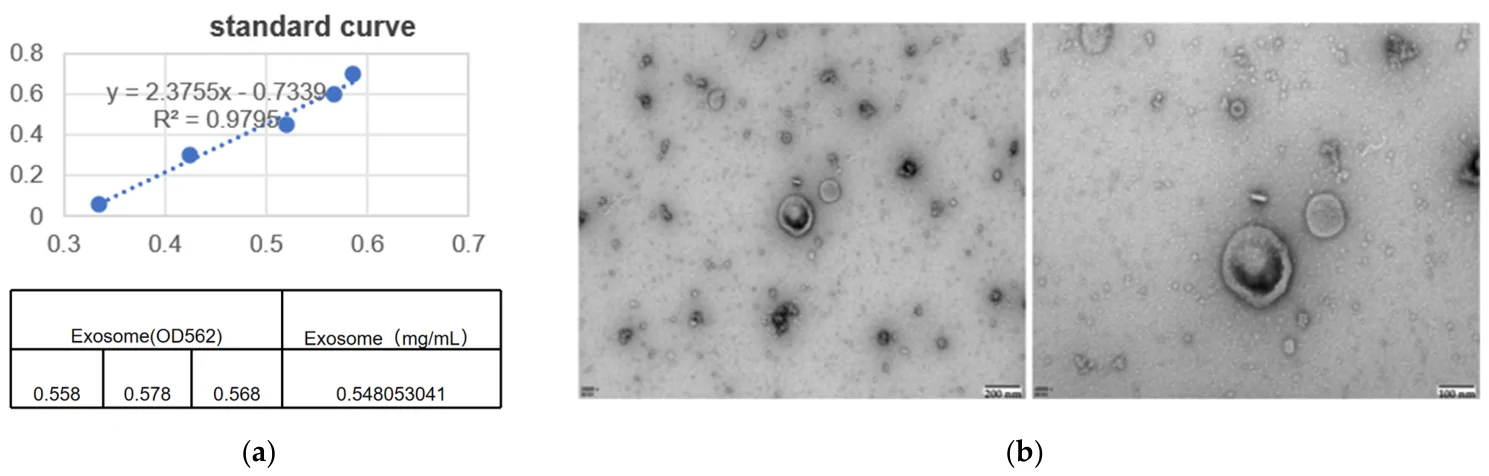

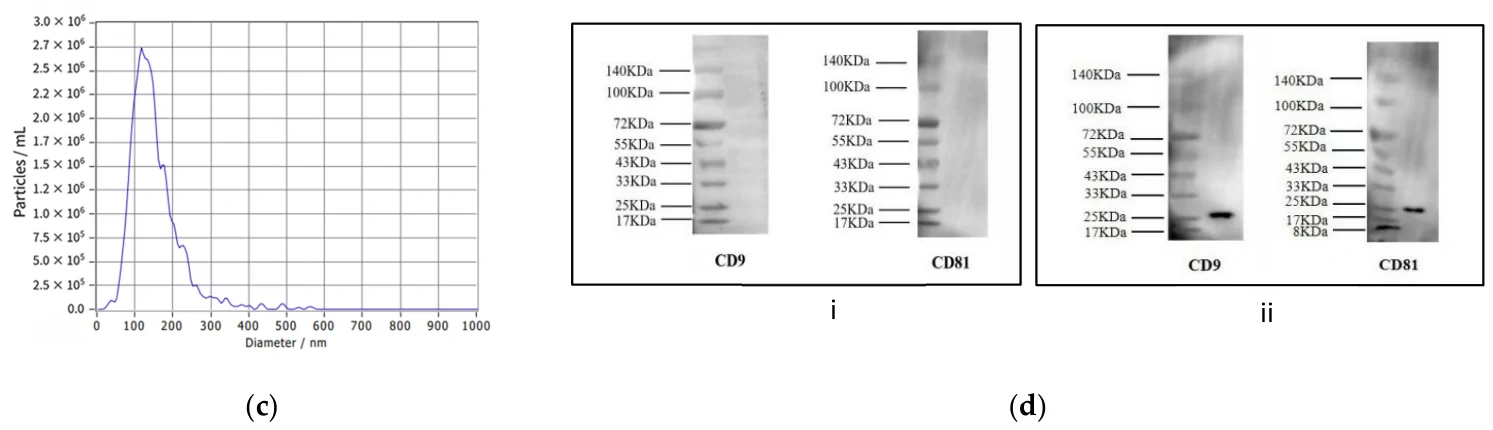
(**a**) The protein concentration of EA-derived EVs. The blue dots indicate the measured OD values of standard protein samples with gradient concentrations, which were used to construct the standard curve for Coomassie Brilliant Blue protein quantification. (**b**) Morphological observation of EA-derived EVs by TEM. (**c**) NTA results of EA-derived EVs. (**d**) WB analysis of CD9/CD81 markers: (**i**) CD9/CD81 expression in EA-derived EVs; (**ii**) CD9/CD81 expression in rat serum exosomes (positive control).

**Figure 2:**
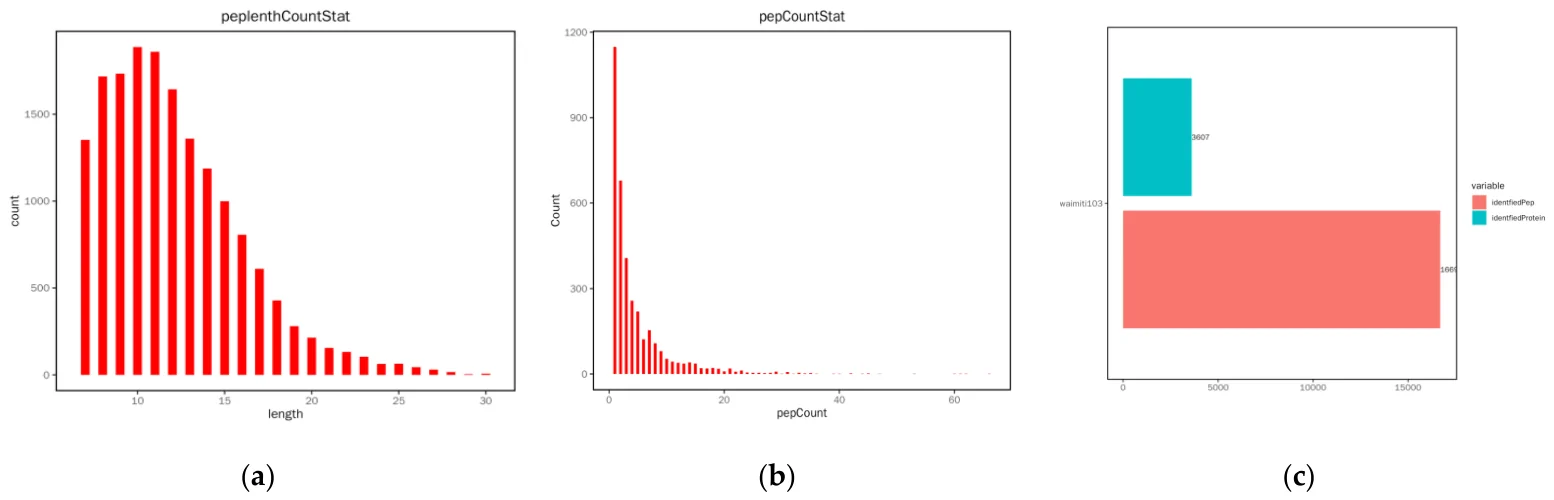
Data of identified peptides. (**a**) Peptide length distribution. (**b**) Number of peptide segments. (**c**) Overview plot of proteins and peptides.

**Figure 3:**
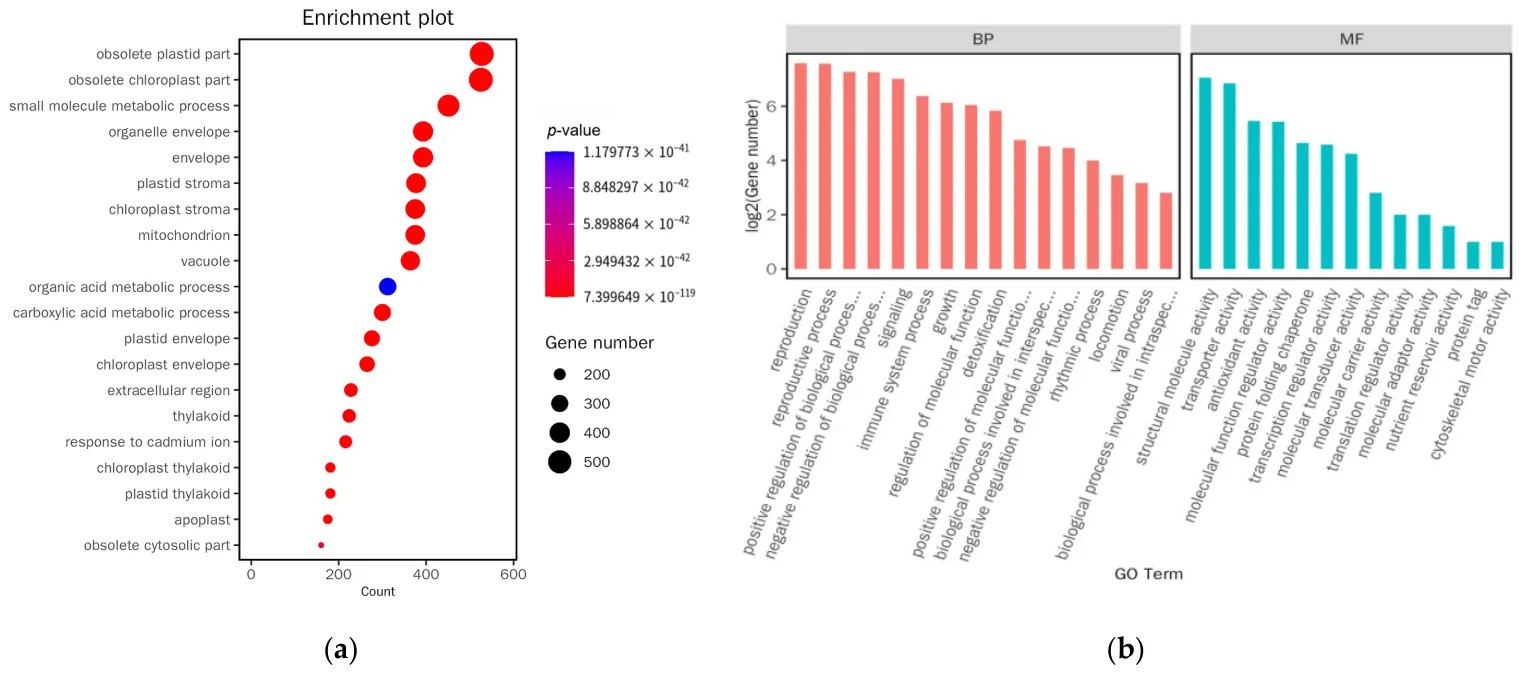
GO enrichment analysis of EA-derived EV proteome. (**a**) Bubble plot of GO functional classification for EA-derived EV proteome. (**b**) WEGO plot of GO functional classification for EA-derived EV proteome.

**Figure 4:**
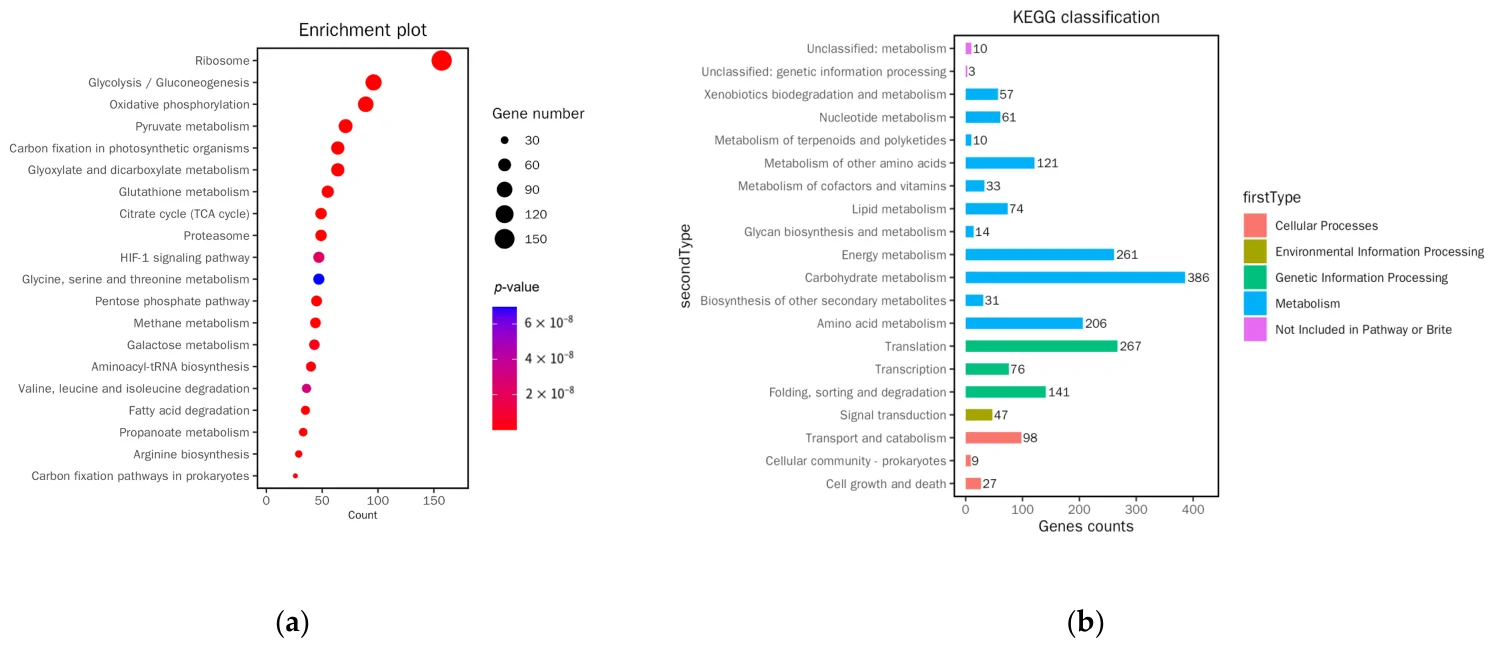
KEGG enrichment analysis of EA-derived EV proteome. (**a**) Bubble plot of KEGG functional classification for EA-derived EV proteome. (**b**) Classification plot of KEGG functional classification for EA-derived EV proteome.

**Figure 5:**
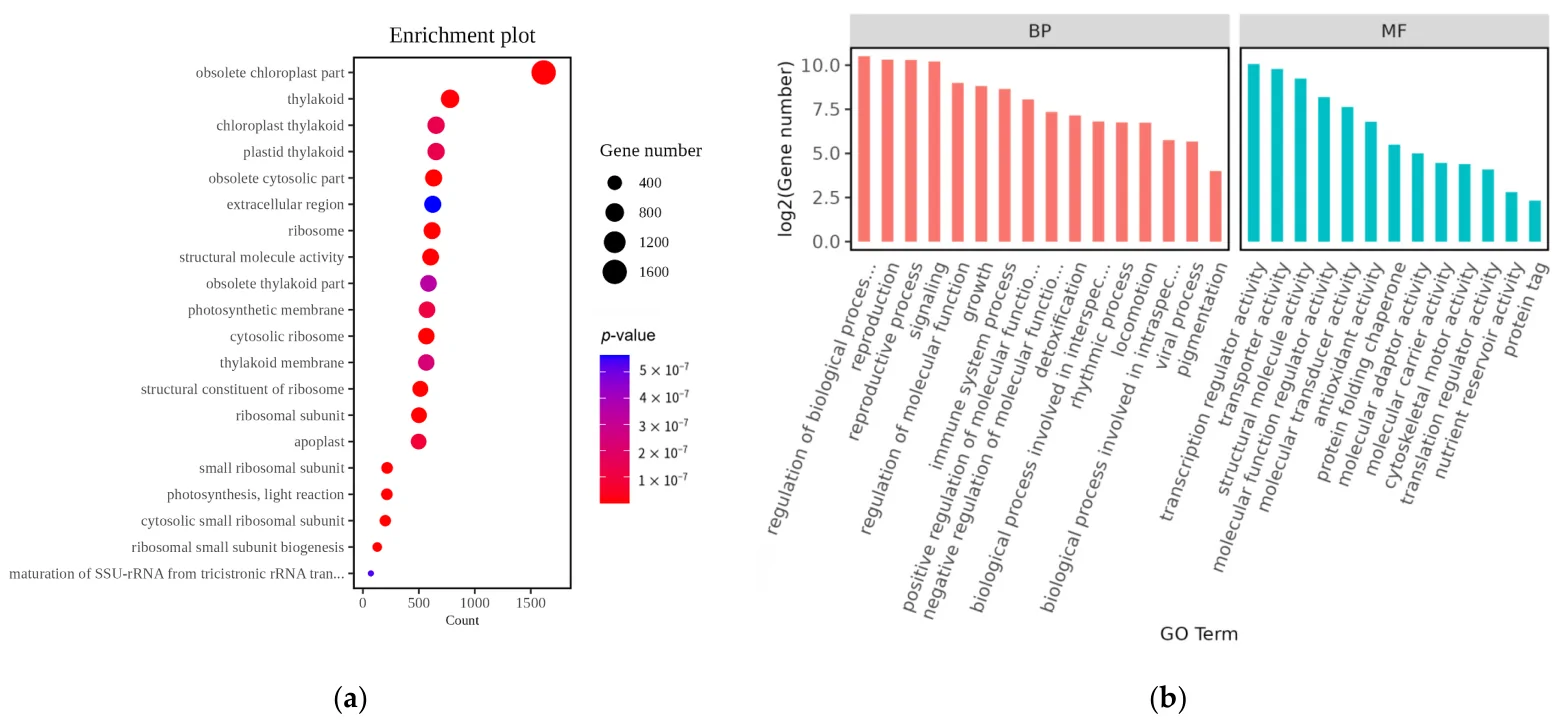
GO enrichment analysis of the identified genes of EA-derived EVs. (**a**) Bubble plot of GO functional classification for EA-derived EV RNA-seq data. (**b**) WEGO plot of GO functional classification for EA-derived EV RNA-seq data.

**Figure 6:**
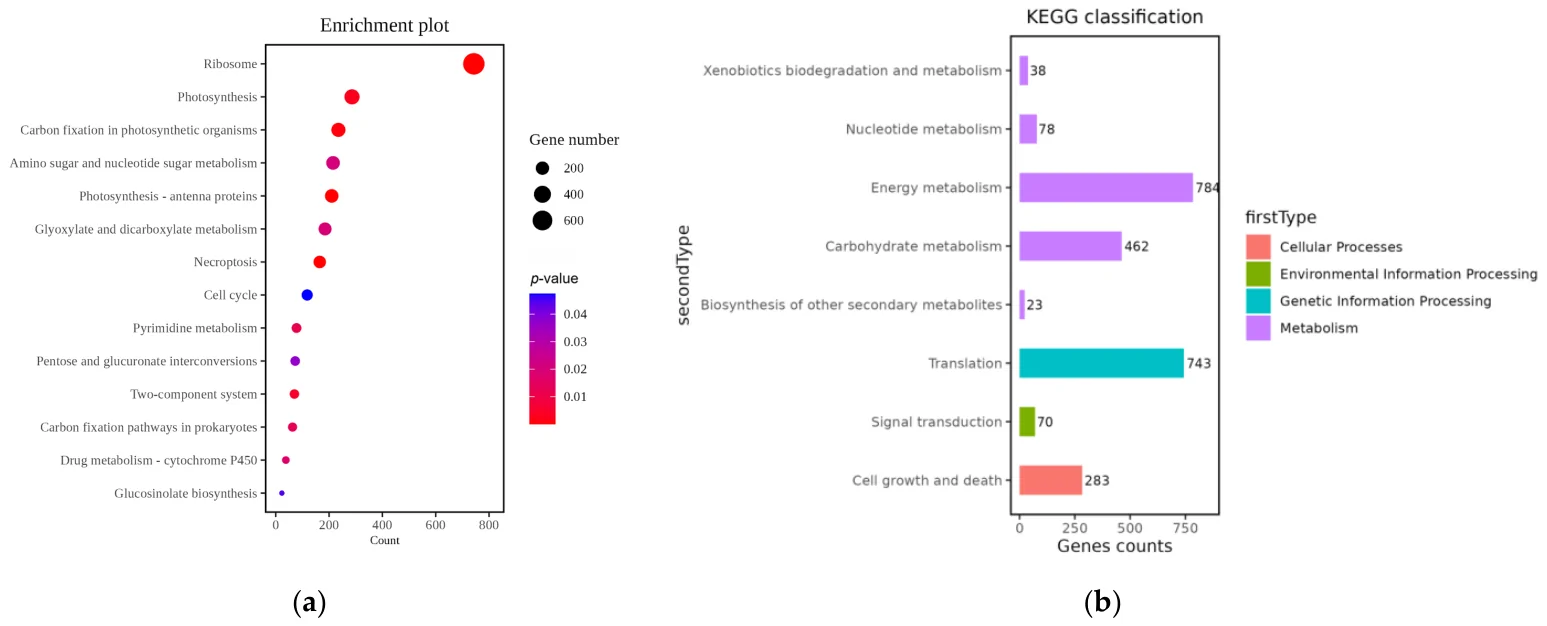
Pathway annotation via KEGG for the identified genes of EA-derived EVs. (**a**) Bubble plot of KEGG functional classification for EA-derived EV RNA-seq data. (**b**) Classification plot of KEGG functional classification for EA-derived EV RNA-seq data.

**Figure 7:**
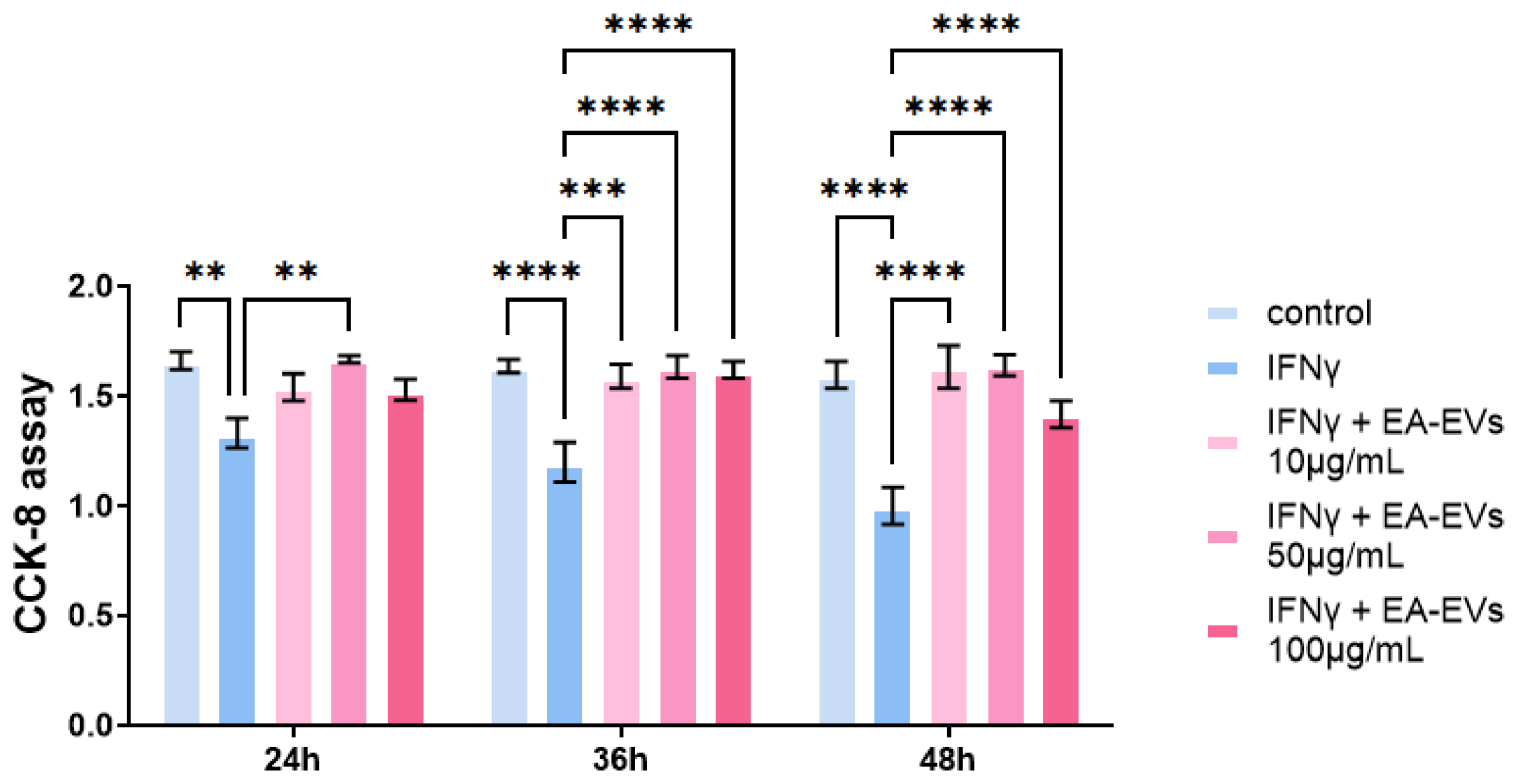
Assessment of cell viability in IFN-γ-stimulated Nthy-ori-3-1 cells following EA-derived EV treatment. ** *p* < 0.01, *** *p* < 0.001, **** *p* < 0.0001.

**Figure 8:**
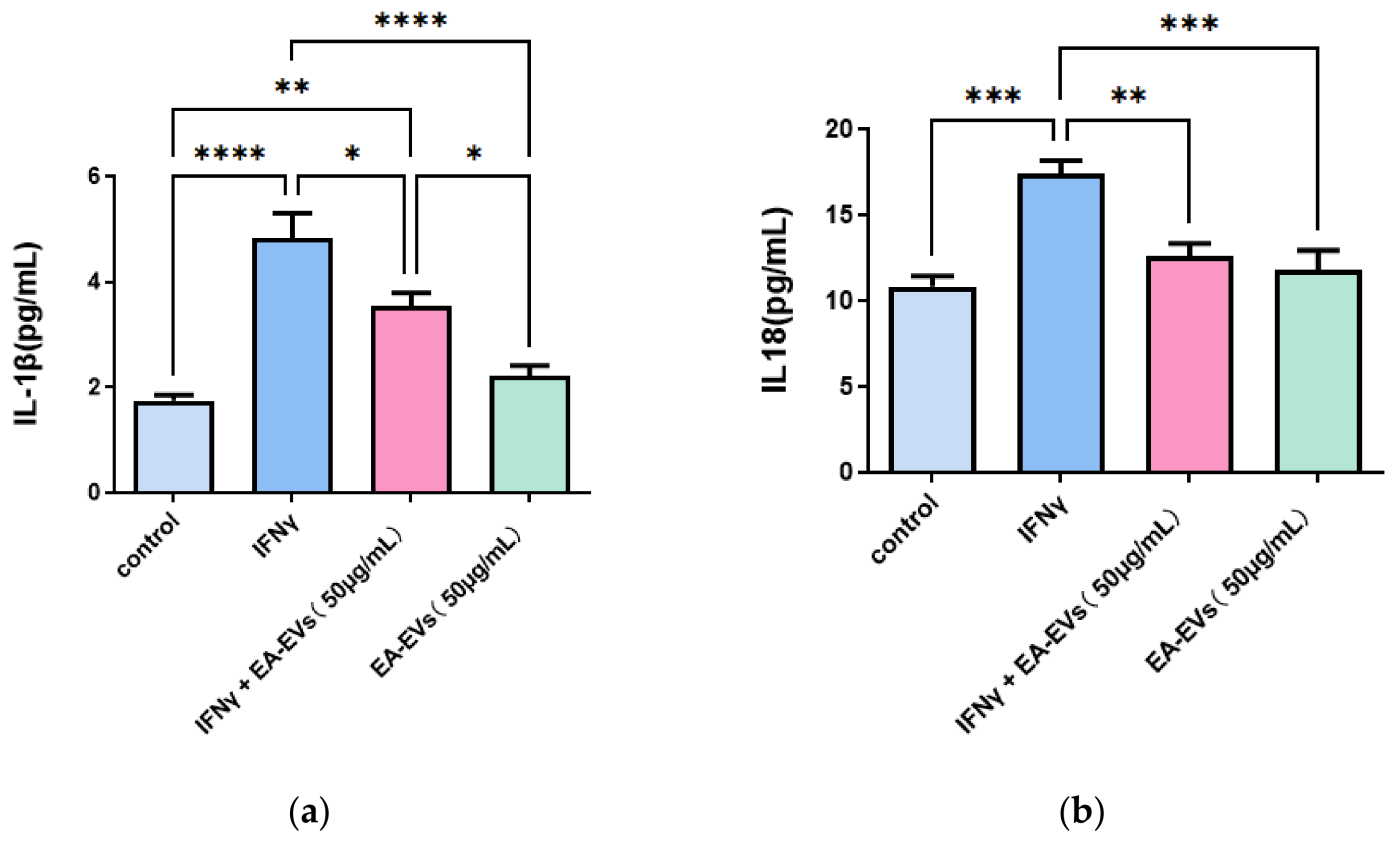
EA-derived EVs inhibit IFN-γ-induced secretion of pro-inflammatory cytokines in Nthy-ori 3-1 cells. (**a**) IL-1β level in supernatant of each group. (**b**) IL18 level in supernatant of each group. * *p* < 0.05, ** *p* < 0.01, *** *p* < 0.001, **** *p* < 0.0001.

**Figure 9:**
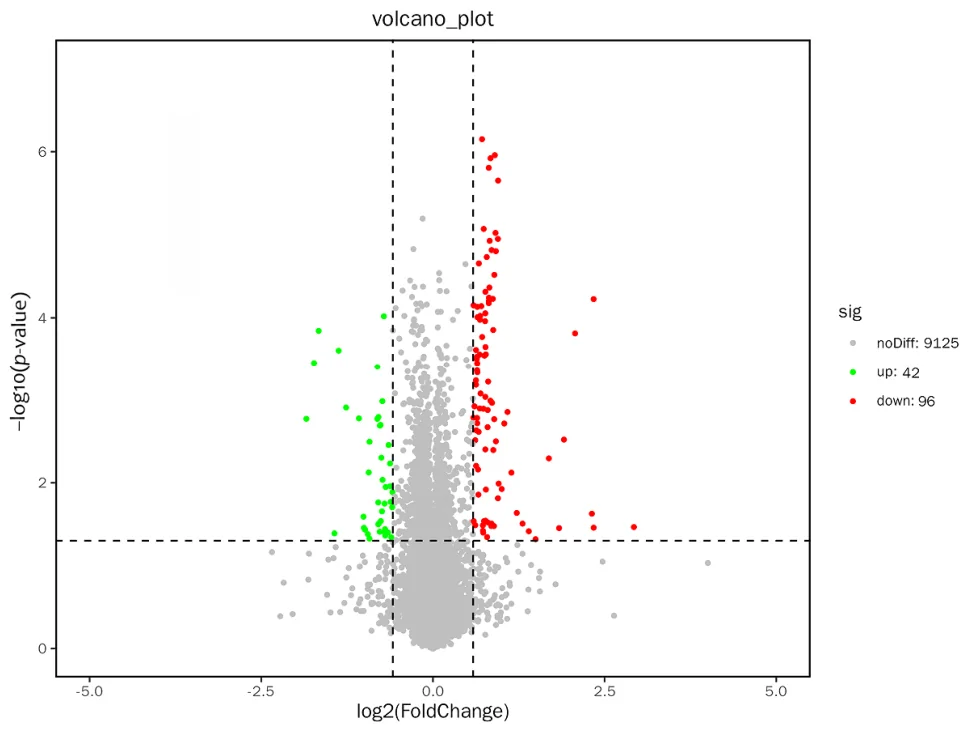
Volcano map depicting differentially expressed proteins between the IFN-γ + EA-EVs and IFN-γ groups.

**Figure 10:**
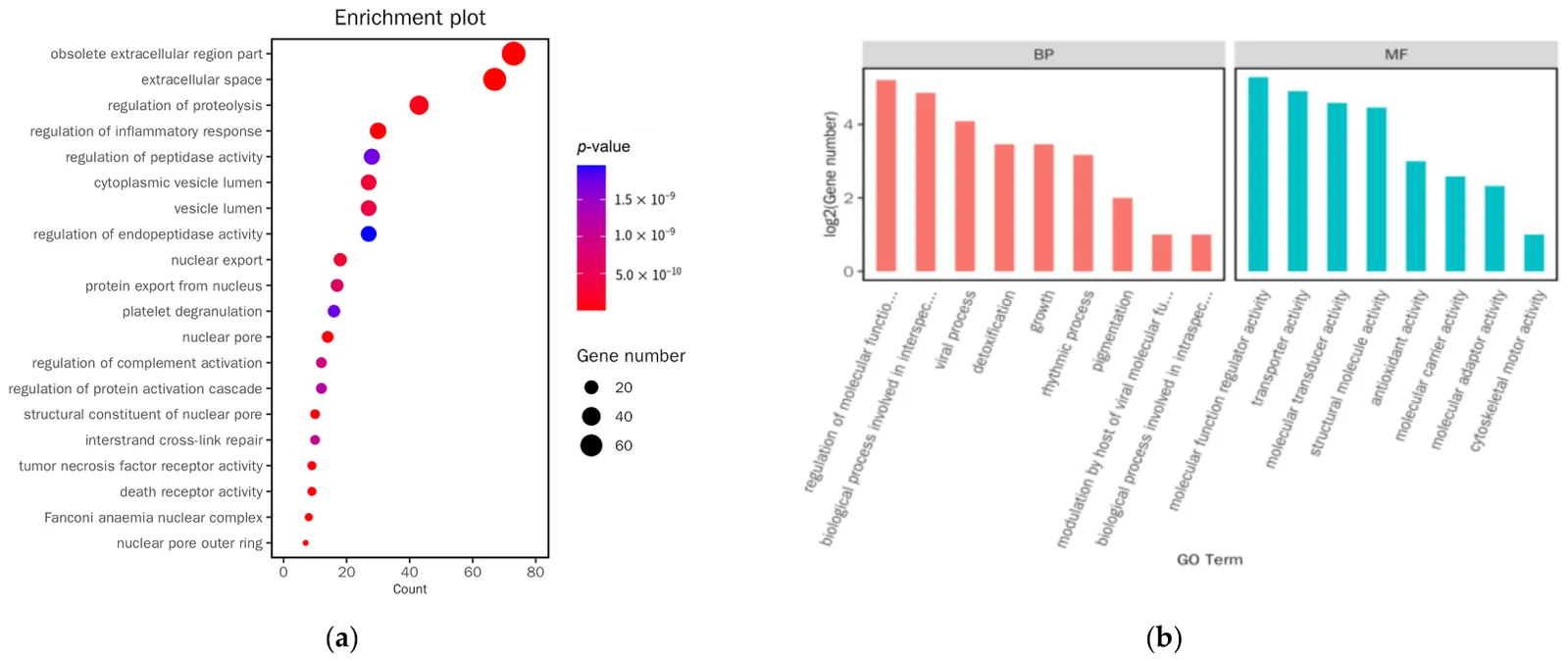

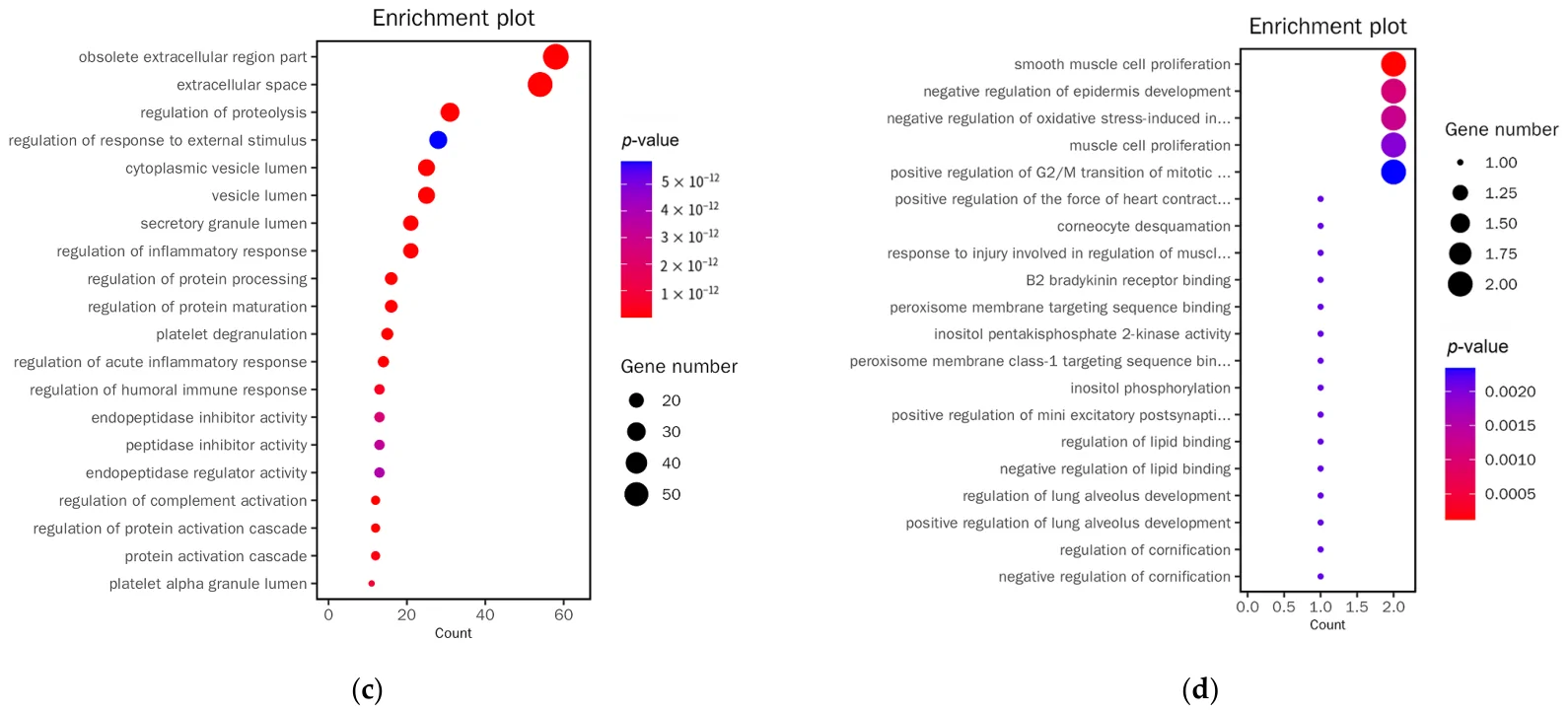
GO functional annotation of the differentially expressed proteins following EV intervention. (**a**) Bubble plot of GO functional classification for differentially expressed proteins (IFN-γ + EA-EVs vs. IFN-γ). (**b**) WEGO plot of GO functional classification for differentially expressed proteins (IFN-γ + EA-EVs vs. IFN-γ). (**c**) Bubble plot of GO functional classification (IFN-γ + EA-EVs vs. IFN-γ, downregulated proteins). (**d**) Bubble plot of GO functional classification (IFN-γ + EA-EVs vs. IFN-γ, upregulated proteins).

**Figure 11:**
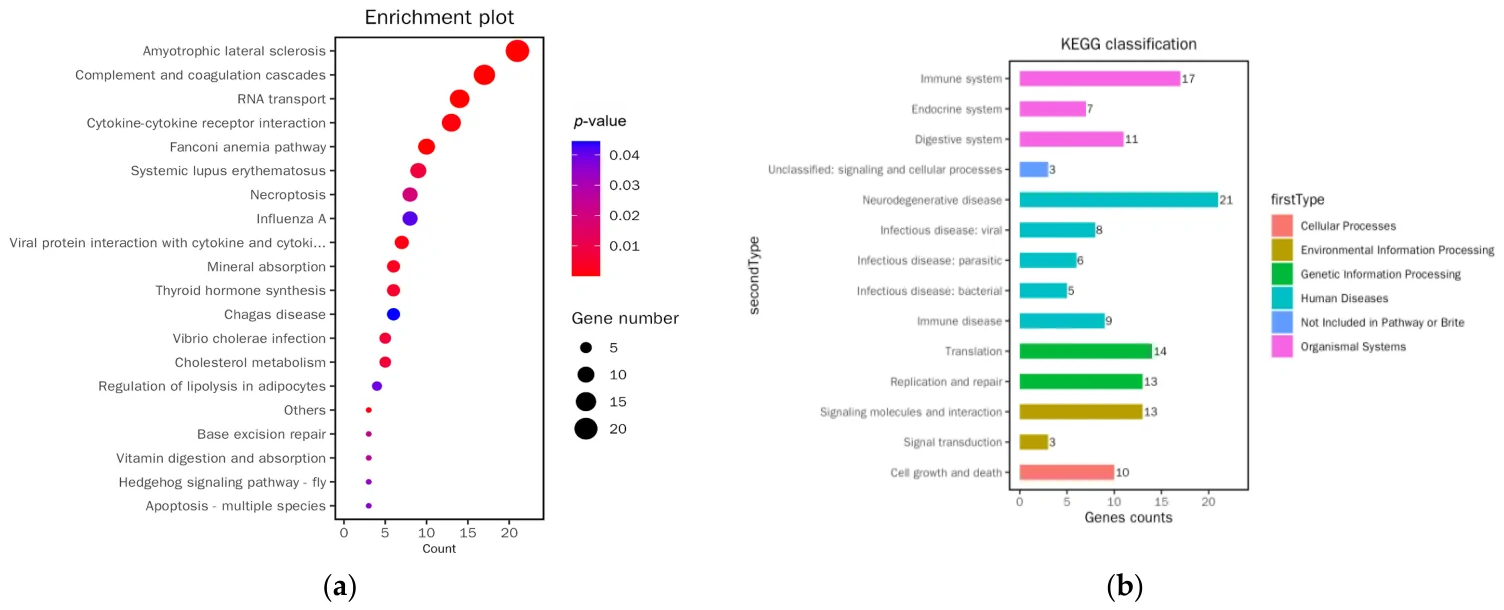

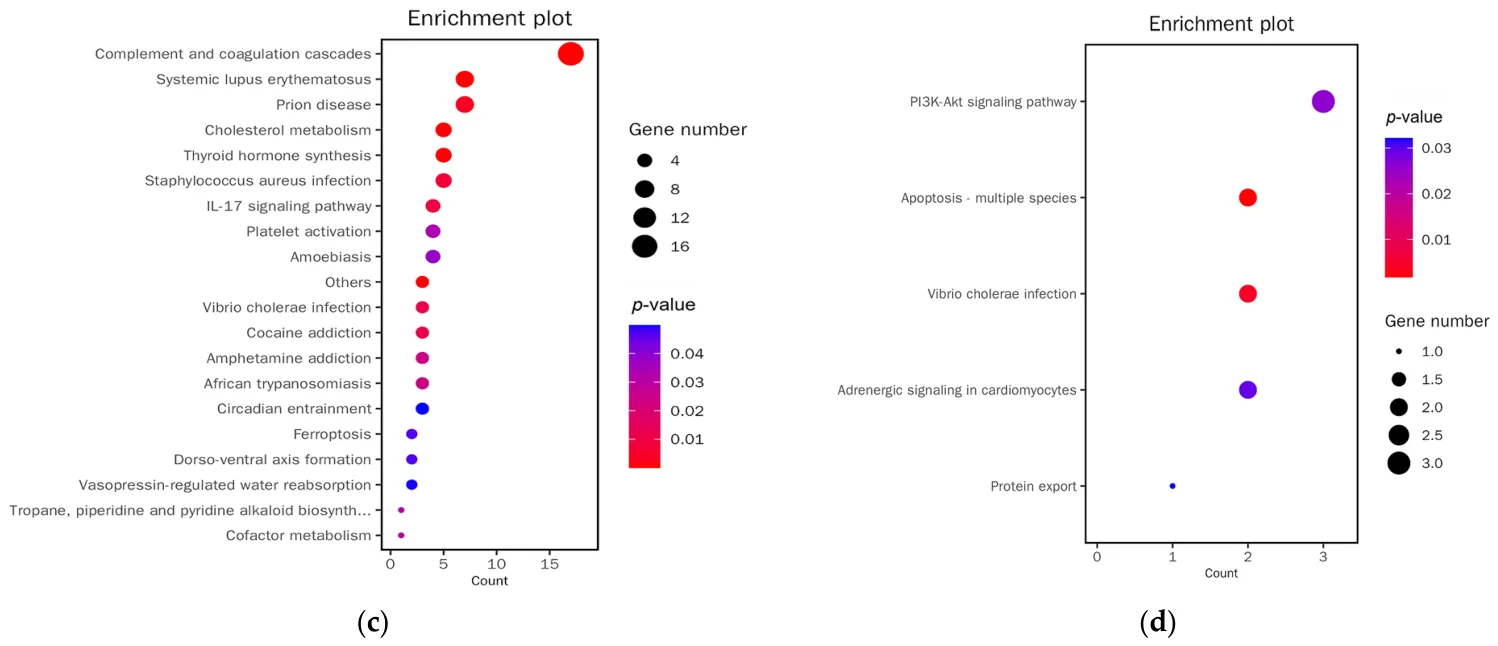
KEGG pathways associated with the EV-modulated proteome. (**a**) Bubble plot of KEGG functional classification for differentially expressed proteins (IFN-γ + EA-EVs vs. IFN-γ). (**b**) Classification plot of KEGG functional classification for differentially expressed proteins (IFN-γ + EA-EVs vs. IFN-γ). (**c**) Bubble plot of KEGG functional classification (IFN-γ + EA-EVs vs. IFN-γ, downregulated proteins). (**d**) Bubble plot of KEGG functional classification (IFN-γ + EA-EVs vs. IFN-γ, upregulated proteins).

**Figure 12:**
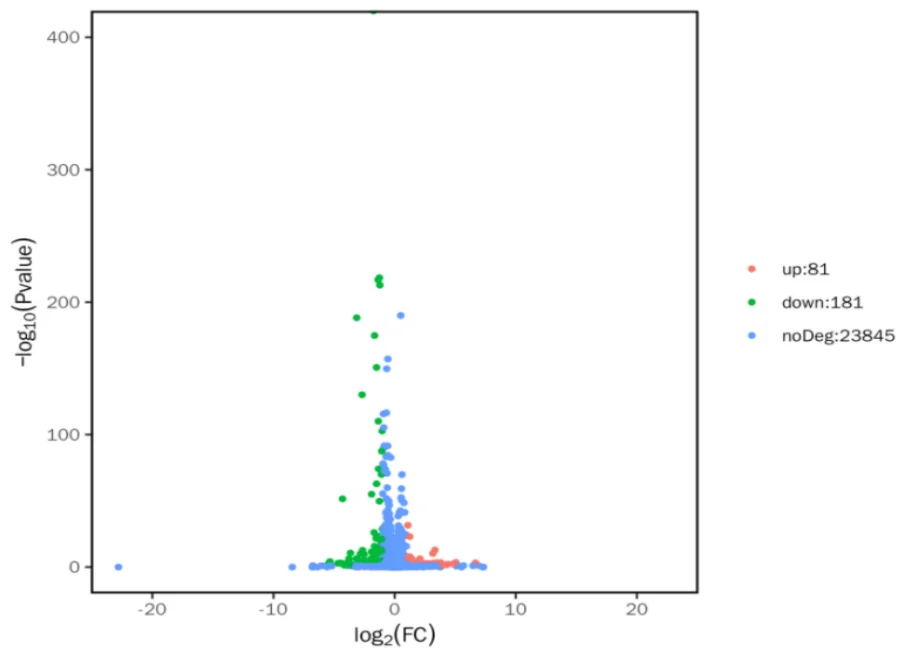
Volcano plot showing the distribution of differentially expressed genes between IFN-γ + EA-EVs and IFN-γ groups.

**Figure 13:**
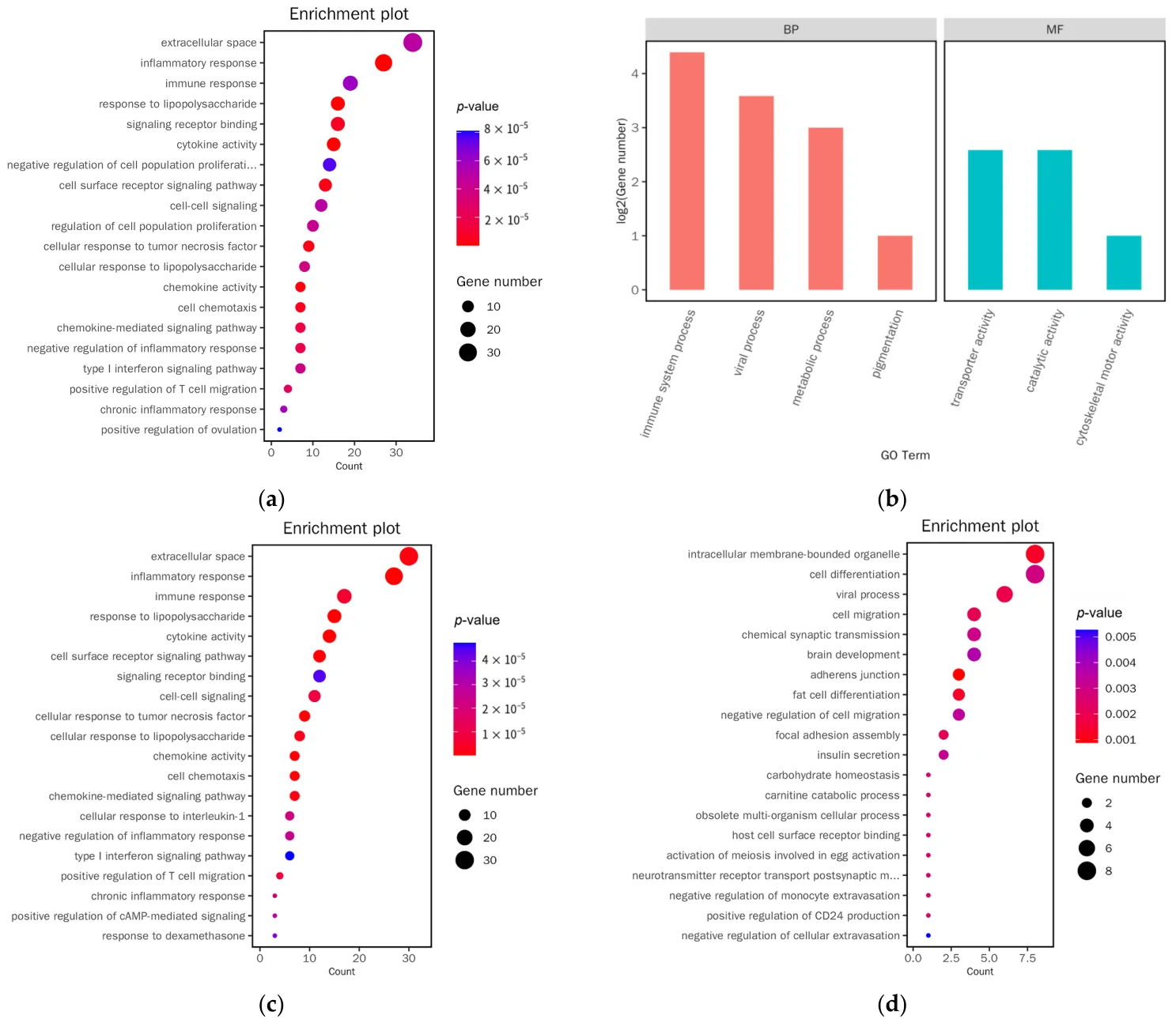
GO term enrichment among the differentially expressed genes under EV treatment. (**a**) Bubble plot of GO functional classification for differentially expressed genes (IFN-γ + EA-EVs vs. IFN-γ). (**b**) WEGO plot of GO functional classification for differentially expressed genes (IFN-γ + EA-EVs vs. IFN-γ). (**c**) Bubble plot of GO functional classification (IFN-γ + EA-EVs vs. IFN-γ, downregulated genes). (**d**) Bubble plot of GO functional classification (IFN-γ + EA-EVs vs. IFN-γ, upregulated genes).

**Figure 14:**
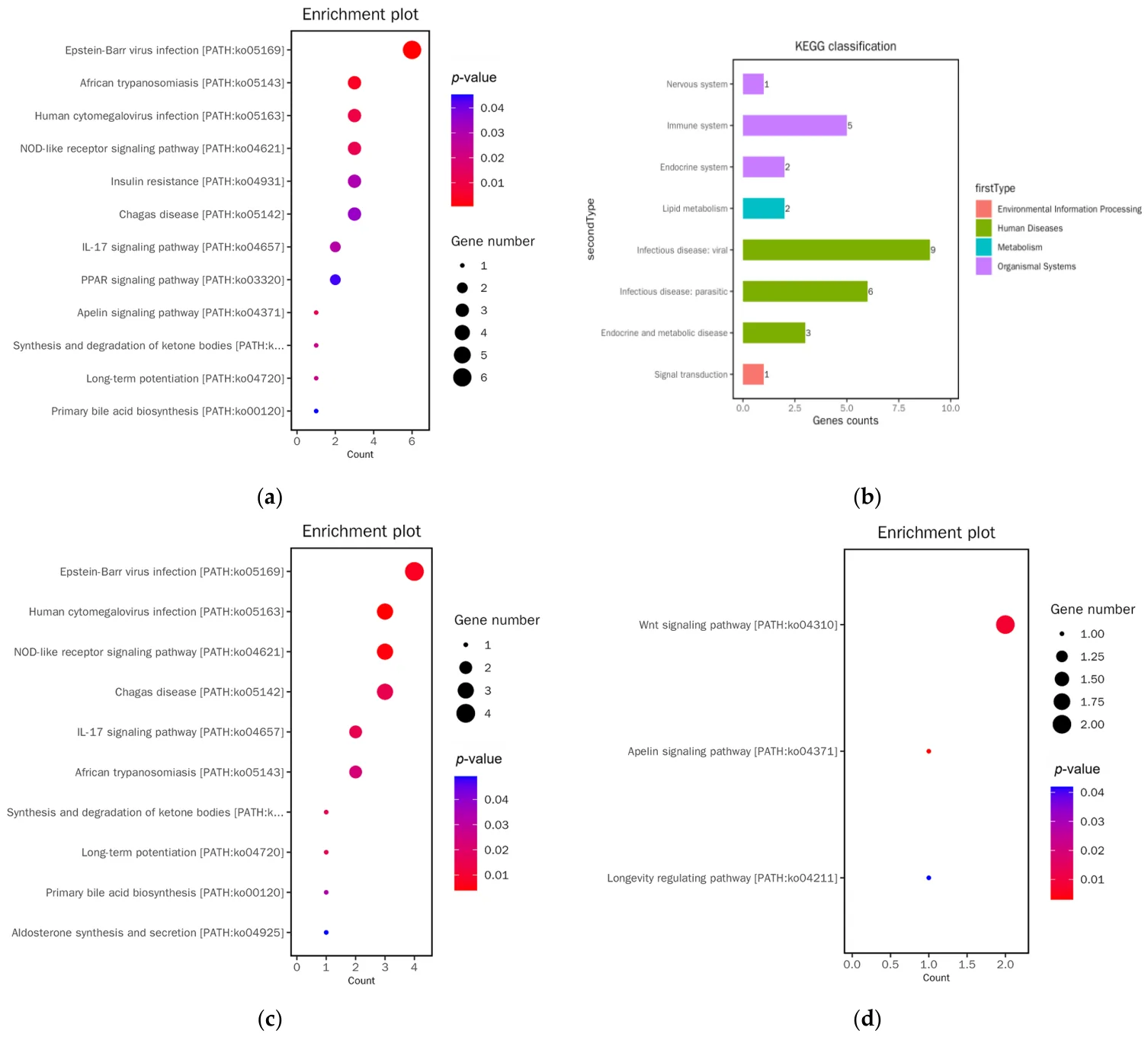
KEGG pathway analysis of genes regulated by EV intervention. (**a**) Bubble plot of KEGG functional classification for differentially expressed genes (IFN-γ + EA-EVs vs. IFN-γ). (**b**) Classification plot of KEGG functional classification for differentially expressed genes (IFN-γ + EA-EVs vs. IFN-γ). (**c**) Bubble plot of KEGG functional classification (IFN-γ + EA-EVs vs. IFN-γ, downregulated genes). (**d**) Bubble plot of KEGG functional classification (IFN-γ + EA-EVs vs. IFN-γ, upregulated genes).

## Data Availability

Data are contained within the article. The original contributions presented in this study are included in the article. Further inquiries can be directed to the corresponding authors.
